# Perioperative Awareness of Spinal Cord Stimulators Amongst Non-consultant Hospital Doctors

**DOI:** 10.7759/cureus.109332

**Published:** 2026-05-21

**Authors:** Harry McGrath, Victoria Giglio, Eoin Cashman, Dominic Harmon

**Affiliations:** 1 Anaesthesia, University Hospital Limerick, Limerick, IRL

**Keywords:** education, knowledge translation, patient safety, perioperative management, spinal cord stimulators

## Abstract

Introduction

Spinal cord stimulators (SCS) are increasingly encountered in surgical patients, requiring specific perioperative management. However, formal training for non-consultant hospital doctors (NCHDs) in Anaesthetics and Pain Medicine, who are meant to specialise in this area, is lacking. This study assessed baseline awareness and the impact of a targeted educational intervention.

Methods

A prospective pre-post study was conducted among NCHDs at a tertiary hospital (n=41). Participants completed surveys before and after a focused teaching session on SCS perioperative management. Outcomes included awareness, knowledge, confidence, and access to specific guidelines, analysed using paired comparisons and normalised effect sizes.

Results

Baseline awareness of SCS presence was high (90%), but only 39% recognised the need for specific management, and 0% had prior training on perioperative management of SCS. Confidence and knowledge of guidance documents were low (41% and 12%, respectively). Post-intervention, all metrics improved significantly: confidence in identifying SCS patients and knowledge of how to access guidance increased to 100% (p<0.001). All participants reported improved understanding, with large to maximal effect sizes across domains.

Conclusion

A significant knowledge deficiency exists in SCS perioperative management among NCHDs. A single targeted educational intervention effectively eliminated this deficit, supporting incorporation into formal training to enhance patient safety.

## Introduction

Spinal cord stimulators (SCS) are implanted neuromodulation devices that deliver electrical impulses to the dorsal columns of the spinal cord, modulating nociceptive signal transmission and thereby reducing the perception of chronic pain [1-3]. They are indicated in the management of chronic pain conditions, including failed back surgery syndrome, complex regional pain syndrome, and refractory angina [1-3]. Worldwide, more than 1.5 billion people experience chronic pain, with low back pain being the most frequently reported pain condition in approximately 25% of the population [3]. SCS has been shown to significantly improve pain relief and quality of life, and therefore, as the use of SCS expands due to evidence and diagnosed chronic pain conditions, the prevalence of patients living with implanted SCS devices continues to rise [1,3,4].

Consequently, the probability that patients with in-situ devices will present for unrelated surgical procedures will rise correspondingly [3,4]. This introduces important perioperative considerations, as SCS systems are complex implantable electronic devices that have the potential for adverse interaction with equipment and monitoring in the perioperative setting. Perioperative management of these patients requires specific knowledge, including device deactivation protocols, intra-operative diathermy precautions, MRI compatibility assessment, and coordination with specialist pain teams [5,6]. Furthermore, the exact type of device from the device manufacturer and anatomic location of the electrode(s) and implantable pulse generator (IPG) should be known, and if this information is not readily available, pre-operative plain film x-rays can be helpful in identifying them [7]. Deactivation of devices immediately prior to the procedure can occur through the patient’s controllers or with assistance from a device representative [8]. Therefore, it is vital to recognise these devices early and plan the perioperative management so as to not delay surgical intervention for these patients.

Despite the growing prevalence and clinical complexity of SCS devices, formal education on their perioperative management has been largely absent from non-consultant hospital doctor (NCHD) training curricula (post-graduate training doctors, below consultant level, ranging from Intern at the most junior to specialist registrar (SpR) at the most senior) [8,9]. There is a recognised gap between the growing prevalence of SCS devices in the general surgical population and the preparedness of frontline junior medical staff to manage these patients safely [6]. A clinician who is unaware of the specific perioperative requirements for SCS patients, or who lacks confidence in managing them, represents a potential risk, including causing a device to overheat, deliver painful stimulation, or change the flow rate in any clinical setting where such patients may present [6]. Therefore, we conducted the current study to evaluate the impact of a targeted educational intervention across perioperative awareness, knowledge, and training on patients with SCS. We also established a quantitative baseline of SCS perioperative awareness by NCHDs at our institution. We investigated pre- vs. post-educational session knowledge and confidence of managing the perioperative patient with an SCS among NCHD clinicians locally, and provided evidence to support national curriculum integration of SCS perioperative management at the NCHD level.

## Materials and methods

Study design

**Figure 1:**
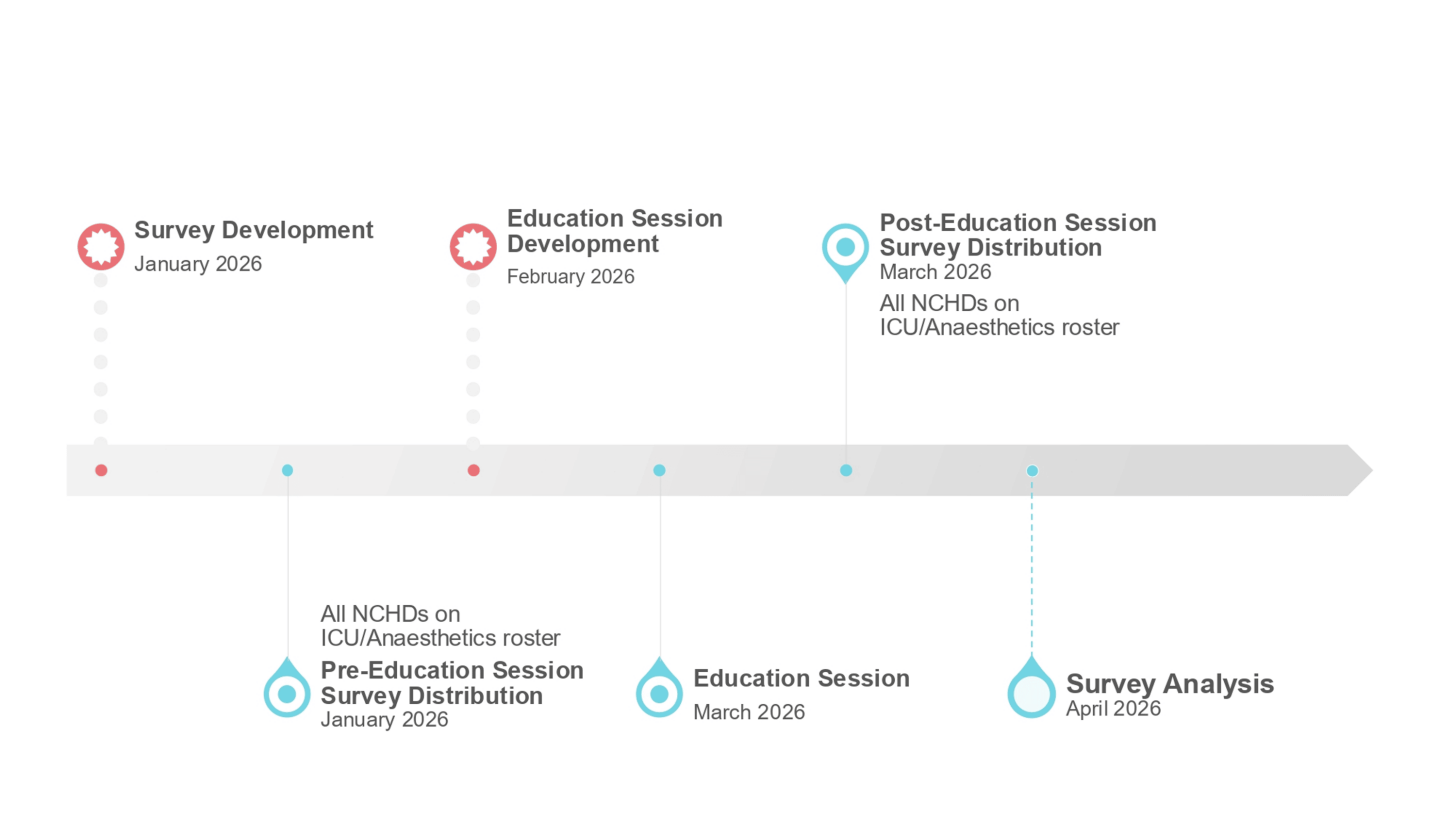
Study design timeline. NCHD: non-consultant hospital doctor, ICU: Intensive Care Unit Image credit: The figure was created by the authors using Microsoft PowerPoint (Microsoft Corp., Redmond, WA, USA), and no AI tools were used in its creation.

This was a prospective pre-post questionnaire study of a single cohort of 44 NCHDs at a tertiary care hospital, University Hospital Limerick, conducted from January 2026 to March 2026 (Figure 1). All participants completed a structured survey immediately before and after a dedicated SCS perioperative education session. Ethics approval was received from the University Hospital Limerick Research Ethics Committee.

Participants

Simple random sampling was applied. All NCHDs, including interns, senior house officers (SHO), registrars, and SpRs on the local ICU/anaesthetic roster, were invited to participate. No exclusion criteria were applied to this population. Participation was voluntary, and consent was implied on submission of the survey. All respondents were actively practising in clinical settings where SCS patients may present perioperatively.

Survey design

A pre-education session survey was developed to assess the perioperative awareness, knowledge, and training of patients with SCS. In this study, “awareness” referred to recognition that patients may present for non-related surgery with an implanted SCS and that these devices require specific perioperative considerations. “Knowledge” referred to self-reported understanding of perioperative management principles, including device identification, device deactivation, diathermy precautions, MRI considerations, and access to relevant SCS perioperative guidelines. “Training” referred to previous formal or informal educational exposure relating to perioperative SCS management. The term “perioperative” was used to specify the context of assessment, namely the preoperative, intraoperative, and immediate postoperative management of patients with SCS devices. The survey was developed by the first author (HM) with review by a consultant anaesthetist (DH) who has expertise in the perioperative management and training of SCS devices. The final survey comprised eight items and consisted of Likert-scale questions [10], binary questions, and brief open-ended questions (Appendix A). A similar post-education survey was developed (Appendix B).

Both surveys were distributed on SurveyMonkey (SurveyMonkey Inc., San Mateo, California, USA), an online survey tool that can save anonymous survey responses. Survey distribution and completion were encouraged at mandatory anaesthetic department NDCH training sessions with daily reminder messages sent out for one week after the survey was initially distributed.

Educational session

An educational session was designed to address the identified competency gaps. The session was based on specialist centre policy documents on SCS and recent literature [5,7]. The session was a one-hour interactive teaching session that included the following sections led by a final-year SpR completing fellowship hours in pain management and an anaesthetics and pain management consultant: (1) Didactic PowerPoint presentation on the use of SCS, including anatomy, technology used, and evidence bases for the use of SCS. This section also included visual pictures of SCS to improve recognition of the same. (2) Didactic PowerPoint presentation on the perioperative management of SCS, including device management protocols, diathermy and electrosurgical precautions, MRI safety considerations, and guidance on accessing perioperative protocols. (3) Case-based discussions, with pictures of SCS to improve recognition, followed by small group discussions on the appropriate perioperative management of the presented cases.

Data analysis

Data were analysed using descriptive statistics and pre-post percentage comparison. McNemar’s test comparing binary outcomes for paired data was used to statistically analyse results pre- and post-intervention using IBM SPSS Statistics for Windows, Version 30 (Released 2024; IBM Corp., Armonk, New York, United States) [11]. Likert responses were converted to a composite score to enable a single numeric comparison across time points: Very Confident = 4, Somewhat Confident = 3, Neutral = 2, Not Confident = 1. Guidance access was scored: Yes = 3, Unsure = 2, No = 1. A composite confidence score was calculated as the mean of all items for each participant. Pre- and post-intervention scores were compared using paired t-tests using IBM SPSS Statistics for Windows [12]. The relative improvement was also expressed as the proportion of the maximum possible gain (normalised gain), calculated as (post-intervention score - pre-intervention score) divided by the maximum achievable improvement (100-baseline) [12]. This provides an intuitive measure of the extent to which potential improvement was realised but does not account for variability.

## Results

Forty-four NCHDs were invited to participate in the survey and training session, with 41 (93%) NCHDs across four training grades completing all 3 components (pre-education survey, education session, post-education survey). The largest group was SHO-grade doctors (n=18, 44%), followed by registrars (n=11, 27%), SpRs (n=9, 22%), and interns (n=3, 7%). The grade distribution is broadly representative of a typical NCHD workforce, with the SpR cohort representing those with the most clinical experience and therefore likely to experience a broad range of perioperative cases (Table 1) [13].

**Table 1:** Training grade distribution (n=41) SCS: spinal cord stimulator, SHO: senior house officer, SpR: specialist registrar

Training Grade	Count (n)	Percentage (%) of Total	Estimated SCS Exposure
Intern	3	7	Low
SHO	18	44	Low-Medium
Registrar	11	27	Medium
SpR	9	22	High
Total	41	100	-

Pre-education survey results

**Figure 2:**
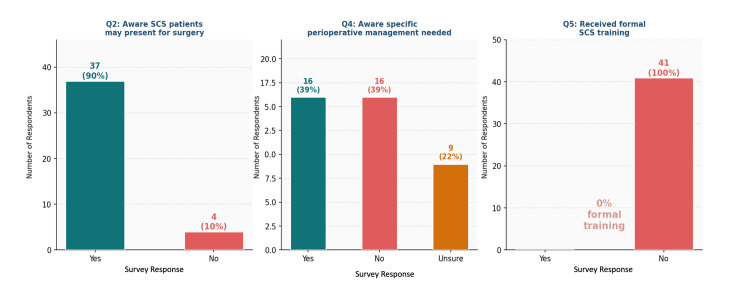
Pre-education awareness and training of patients reported by all survey respondents (n=41) with spinal cord stimulators (SCS). Image credit: The figure was created by the authors using Microsoft Excel (Microsoft Corp., Redmond, WA, USA).

The majority of respondents (37/41, 90%) were aware that patients with implanted SCS devices can present for surgery; however, only 16/41 (39%) were aware that SCS devices require specific perioperative management. No respondents had received formal training on this topic (Figure 2 and Table 2).

**Table 2:** Training grade exposure to spinal cord stimulators (SCS) training (n=41). SHO: senior house officer, SpR: specialist registrar

Grade	n	Untrained (n)	Untrained (%)	SCS Exposure	Estimated Risk
Intern	3	3	100	Low	Low current risk; foundational awareness gap
SHO	18	18	100	Low-Medium	Most numerous grade; widest impact due to NCHD volume
Registrar	11	11	100	Medium	Growing decision-making role
SpR	9	9	100	High	Senior decision-maker - highest risk
Total	41	41	100	-	Universal deficit in perioperative SCS training

**Figure 3:**
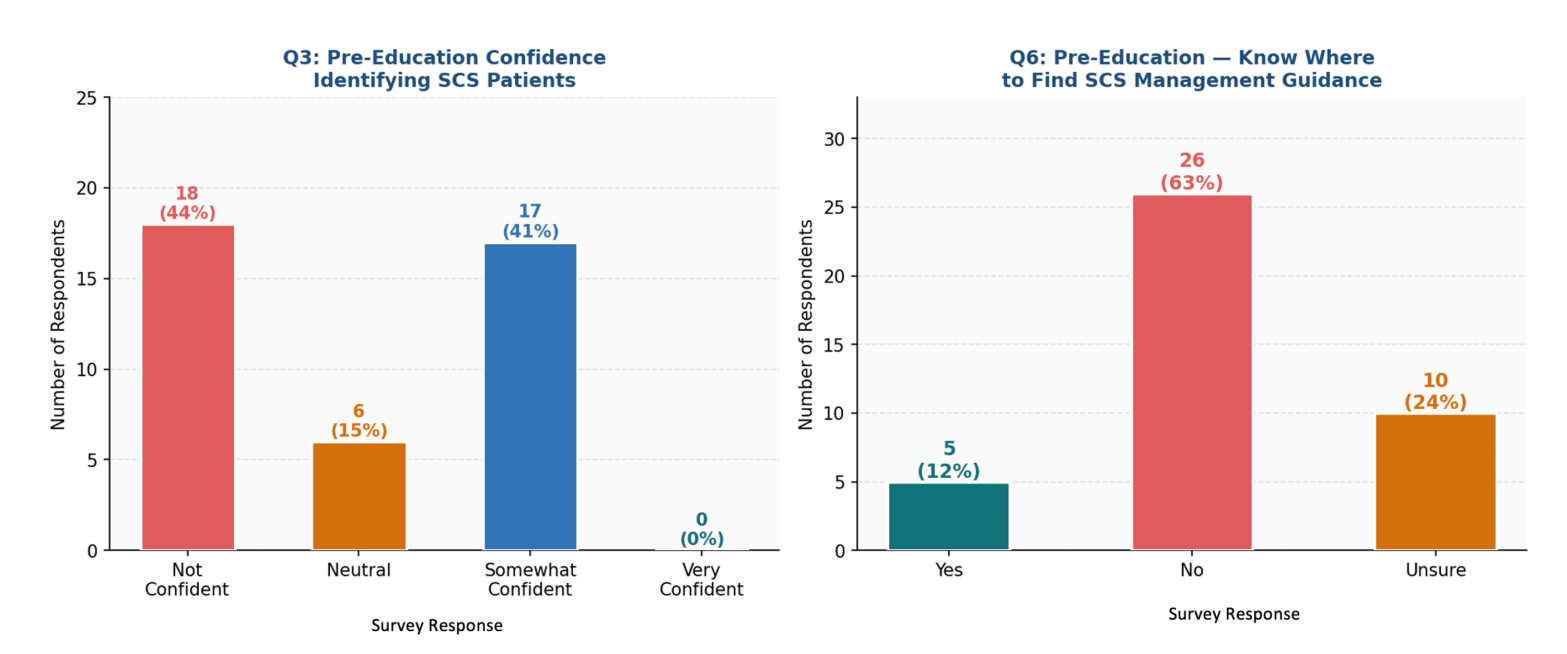
Pre-education confidence in identifying patients with spinal cord stimulators (SCS) (Question 3, left) and knowledge of access to guidance (Question 6, right) among all survey respondents (n=41). Image credit: The figure was created by the authors using Microsoft Excel (Microsoft Corp., Redmond, WA, USA).

Self-reported confidence in identifying a patient with an SCS device on examination during pre-operative assessment was predominantly low: 18/41 (44%) reported being 'Not Confident', 6/41 (15%) were Neutral, and 17/41 (41%) were 'Somewhat Confident'. No respondents selected 'Very Confident'. Access to perioperative guidelines of SCS was also low: only 5/41 (12%) knew where to find it, whilst 26/41 (63%) stated they would not know where to look (Figure 3).

Despite the clear knowledge deficit, motivation to learn was high. Thirty-six participants (88%) stated they would be interested in further teaching or guidelines on SCS perioperative management, with 5/41 (12%) responding 'Maybe' and zero respondents declining.

Post-education survey results

Post-education, confidence in identifying SCS patients was divided between 'Very Confident' (21/41, 51%) and 'Somewhat Confident' (20/41, 49%), with no respondents answering Neutral or Not Confident. Self-reported confidence in perioperative management of these patients was similarly high, with 8/41 (44%) reporting ‘Very Confident’ and 23/41 (56%) ‘Somewhat Confident’. All respondent reported that their understanding improved, with 22/41 (54%) describing it as significantly improved.

**Figure 4:**
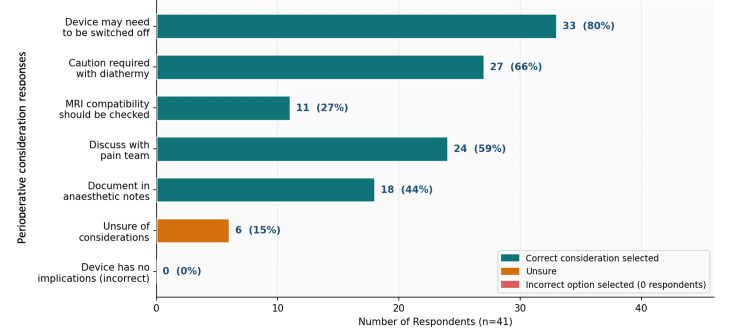
Post-education perioperative knowledge of spinal cord stimulators (respondents could select all that applied) reported by all survey respondents (n=41). Image credit: The figure was created by the authors using Microsoft Excel (Microsoft Corp., Redmond, WA, USA).

When asked to identify important perioperative considerations, respondents could select all that they thought were important. The most commonly selected correct answers were: device may need to be switched off prior to surgery (33/41, 80%), caution required with diathermy (27/41, 66%), and discussion with the pain team (24/41, 59%). Notably, no respondents selected the incorrect option ('device has no implications') (Figure 4).

Eighty-three per cent of respondents (34/41) believe that formal teaching on SCS should be included in NCHD education or induction programmes, with 17% unsure and none opposed.

Pre- vs. post-education session analysis

All metrics, including awareness, confidence, knowledge, access to guidance, and overall impact of teaching of SCS, showed marked improvement following the education session.

Regarding confidence in identifying patients with SCS, there was a statistically significant change in paired responses following the intervention, with 41% of respondents somewhat or very confident pre-session compared with 100% of respondents post-session (p<0.001). Regarding accessing guidance on perioperative management of SCS, there was a statistically significant change in paired responses following the intervention, with 12% of respondents knowing where to find SCS guidance pre-session compared with 100% of respondents post-session (p<0.001).

**Figure 5:**
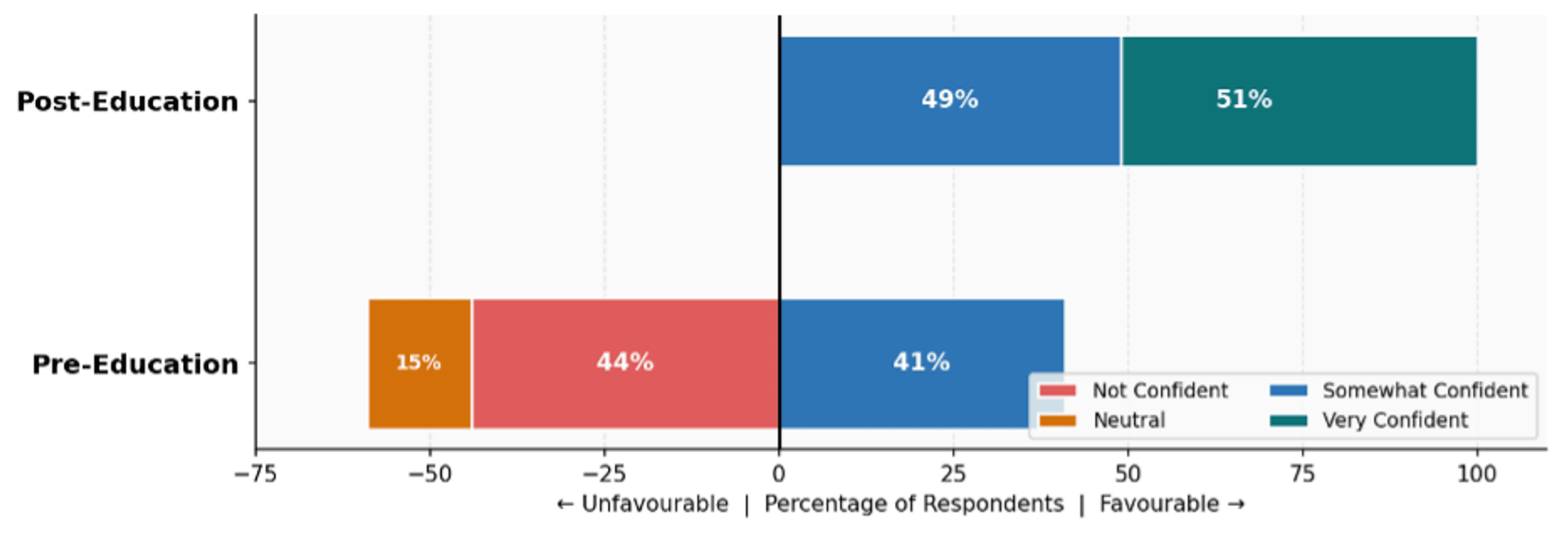
Diverging Likert chart showing confidence in the perioperative management of patients with spinal cord stimulators reported by all survey respondents (n=41). The negative mass (left of centre) was fully eliminated post-education. Image credit: The figure was created by the authors using Microsoft Excel (Microsoft Corp., Redmond, WA, USA).

The diverging Likert chart provides a striking visual of the shift in confidence. Pre-education, the bar extends 59 percentage points (pp) into negative territory (Not Confident 44% + Neutral 15%). Post-education, the entire bar lies in positive territory (Somewhat 49% + Very Confident 51%), representing the complete elimination of all unfavourable confidence responses (Figure 5).

**Figure 6:**
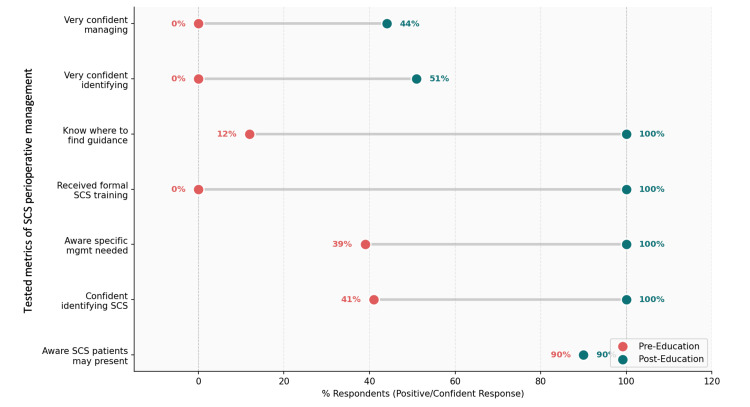
Dumbbell chart of pre-education and post-education survey responses (n=41). Coral: pre-intervention, Teal: post-intervention, SCS: spinal cord stimulator Image credit: The figure was created by the authors using Microsoft Excel (Microsoft Corp., Redmond, WA, USA).

All metrics were simultaneously compared descriptively using a dumbbell plot. The dumbbell plot illustrates the before-and-after shift for every metric simultaneously. Access to SCS guidance recorded the largest single shift (+88pp), followed by management awareness (+61pp) and confidence in identifying patients (+59pp). Awareness of patients presenting with SCS recorded no change (0pp), reflecting a pre-existing awareness that was already high. Every metric either improved or held at its existing level. Of note, not a single domain declined (Figure 6).

**Figure 7:**
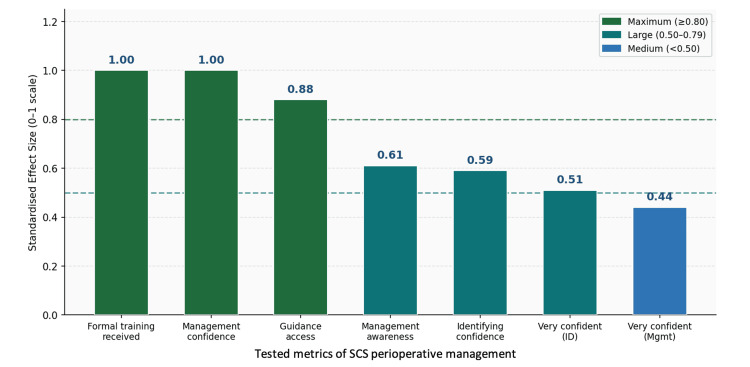
Relative improvement size per domain (0=no effect; 1.00=maximum possible; reference lines at 0.50 and 0.80) (n=41). SCS: spinal cord stimulator Image credit: The figure was created by the authors using Microsoft Excel (Microsoft Corp., Redmond, WA, USA).

All metrics showed an improvement in normalised gain post the educational session. By conventional benchmarks, all metrics but one showed 'large' (≥0.50) or 'maximum' improvements, confirming that the educational intervention was not merely statistically significant but clinically meaningful across every domain (Figure 7).

The Certainty Index separates 'definitive' answers (Yes/No/Very Confident) from hedged responses (Unsure/Neutral/Maybe). Pre-education, 22-24% of respondents were uncertain about management and guidance. Post-education, uncertainty was effectively eliminated across all clinical competency questions. The only residual uncertainty (17%) related to the curriculum question, which reflects an opinion rather than a clinical knowledge deficit.

**Figure 8:**
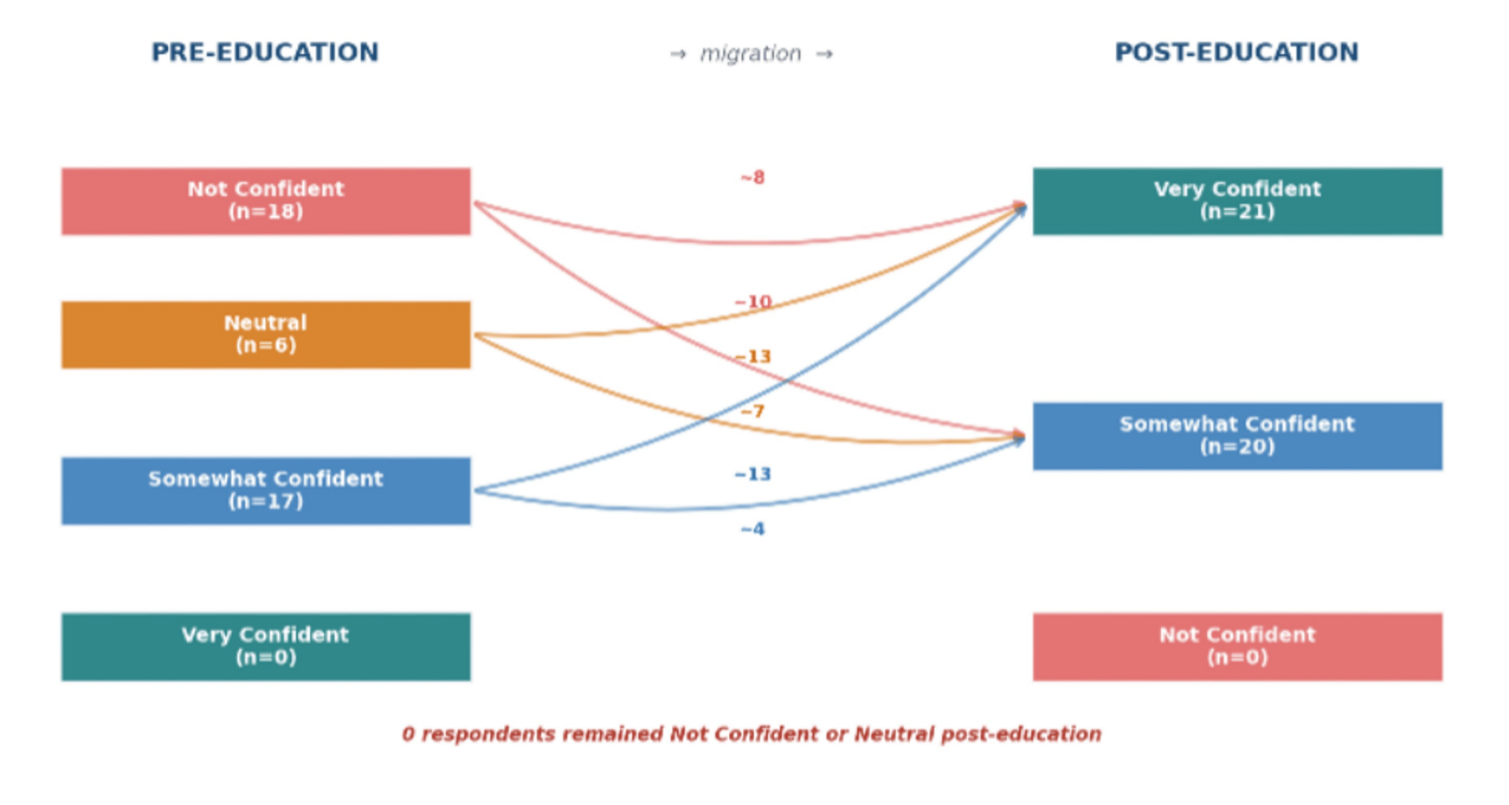
Response migration illustrating how pre-education confidence categories redistributed post-education (approximate flows) (n=41). Image credit: The figure was created by the authors using Microsoft Excel (Microsoft Corp., Redmond, WA, USA).

Lastly, the individual level shift in clinical confidence was also assessed. The response migration chart traces how respondents from each pre-education confidence category redistributed post-education. This analysis moves beyond aggregate percentages to show the individual-level shift in clinical confidence, demonstrating improvement in confidence in managing patients with SCS perioperatively. Of the 18 respondents who were 'Not Confident' pre-education, approximately eight moved to 'Very Confident' and 10 to 'Somewhat Confident'. Of the 17 who were 'Somewhat Confident', approximately 13 moved to 'Very Confident'. The six Neutral respondents were redistributed across both positive categories. Zero respondents remained in any negative confidence category (not confident or neutral) after education (Figure 8).

Table 3 summarises all key metrics across both surveys, with pre-education risk classification, pp gain, and magnitude band.

**Table 3:** Summary of all key metrics before and after the education session, showing the raw percentage point (pp) gain in improvement across all domains (n=41). SCS: spinal cord stimulators, NCHD: non-consultant hospital doctor

Competency/Question	Pre (%)	Post (%)	Gain	Pre-risk	Notes
Received formal SCS training	0	100	+100pp	High	Critical gap - universal deficit fully closed
Confident in SCS management	0	100	+100pp	High	All respondents are confident post-education
Knows where to find guidance	12	100	+88pp	High	Near-total gap resolved
Understanding is improved by teaching	0	100	+100pp	High	100% report improvement
Confidently identifying SCS patients	41	100	+59pp	Medium	Includes somewhat confident
Aware that specific management is needed	39	100	+61pp	Medium	Major knowledge deficit pre-education
Very confident in identifying SCS	0	51	+51pp	Medium	0% → 51% at highest confidence tier
Very confident managing SCS	0	44	+44pp	Medium	0% → 44% at highest confidence tier
SCS teaching in the NCHD curriculum	-	83	-	-	83% support post-education (none opposed)
Aware SCS patients may present	90	90	0pp	Low	Pre-existing awareness - no change

## Discussion

The findings of this study demonstrate a baseline deficit in SCS perioperative knowledge across all NCHD grades, followed by drastic improvement of this deficit after a targeted educational intervention. This magnitude of change is unusual in medical education literature, where improvements are typically modest rather than universal [14,15]. The scale of improvement observed here likely reflects the depth of the baseline knowledge gap, the focused, clinically relevant nature of the intervention, and local focus on knowledge acquisition and engagement in education curated within our Department of Anaesthesiology.

The universal absence of formal SCS training identified in this study alludes to a potentially broader issue within postgraduate medical education in Anaesthesiology and Pain Medicine. Despite clear guidelines outlining safe perioperative management of neuromodulation devices, these are not consistently incorporated into training curricula [5,6]. This creates a scenario in which clinicians may encounter patients with implanted devices without the necessary knowledge to manage them safely. Errors in SCS perioperative management, including failure to deactivate the device, inappropriate use of diathermy, or failure to assess MRI compatibility, are well-described and can result in device malfunction, neurological injury, or permanent hardware damage [5,6,16]. The elimination of these knowledge deficits following a single educational intervention demonstrates the effectiveness of targeted education in mitigating them.

The observed improvements align with established models of medical education, particularly those demonstrating that targeted, problem-focused teaching produces greater behavioural and knowledge change than passive or general educational approaches [8,14]. The transition of respondents from 'not confident' to 'very confident' within a single session suggests that the barrier to competency was structural (absence of curriculum provision) rather than experiential.

In clinical contexts, ambiguity and uncertainty may adversely affect decision-making, contributing to indecision, delayed action, or failure to seek appropriate support [17]. Educational interventions that reduce uncertainty, therefore, have a direct patient safety benefit. Therefore, the elimination of uncertainty (neutral or unsure responses) across all clinical domains seen in our study is significant in two ways. First, the movement from 'Not Confident' to 'Very Confident' in a single session demonstrates the effectiveness of targeted, competency-focused education over generic awareness-raising. Second, the complete elimination of the Neutral and Not Confident categories suggests that the post-education cohort is uniformly functional, there are no residual pockets of clinical uncertainty, and it offers the opportunity to improve physician growth with a targeted educational intervention.

The SpR cohort, despite representing only 22% of respondents, carries the highest clinical responsibility among NCHDs in perioperative settings and is expected to discuss a perioperative management plan with their consultant supervisor when an SCS patient presents. The finding that this cohort shared the same zero-training baseline as interns is among the most clinically significant observations of this study. The strong support among respondents for formal inclusion of SCS education (83%) is consistent with literature demonstrating that clinicians value training that directly improves clinical competence and patient safety [9]. Given the increasing prevalence of neuromodulation devices, incorporation of SCS management into NCHD induction and ongoing training programmes is both justified and necessary.

Limitations

The design of this study allows direct within-cohort comparison while controlling for inter-individual variation. However, the lack of a control group represents an important limitation. In this single-cohort pre-post design, causal attribution relies on temporal association, and residual confounding cannot be excluded. Moreover, this study only evaluated a single centre cohort of anaesthetic and ICU NCHDs and may not reflect knowledge, awareness, and confidence among NCHDs at other centres. Furthermore, the results of this study describe short-term outcome measures, as the post-education survey was distributed immediately after the education session. Further investigation of knowledge retention at three, six, and 12 months would be beneficial to ensure trainees retain the benefits of educational sessions. Lastly, the results of our study reflect perceived competence rather than objectively assessed clinical performance, which may further be biased by respondents' awareness that they had just received teaching. Objective skills assessment, specific knowledge questions of perioperative management of SCS, or an audit of perioperative anaesthetic records of patients presenting with SCS would strengthen the evidence base.

Future directions

The findings of this study have direct patient safety implications. A clinician who is unaware of the specific perioperative requirements for SCS patients, or who lacks confidence in managing them, represents a potential risk in any clinical setting where such patients may present. The results from this study have led to the development of a mandatory SCS perioperative management training module in NCHD induction programmes at all grades that aligns with a local, accessible perioperative SCS guideline. This study will be replicated across all professional grades who encounter SCS in the perioperative period. It is aimed to conduct a follow-up survey to assess knowledge retention following the initial session. Furthermore, we will expand the study to other hospital sites and clinical specialities to establish the generalisability of findings and engage with national postgraduate training bodies (e.g., the Royal College of Surgeons in Ireland (RCSI), Irish Medical Council) to pursue formal curriculum inclusion. It is envisaged to conduct a prospective clinical audit to evaluate whether competency gains translate to measurable improvements in SCS patient outcomes. This may include the development of train-the-trainer resources to enable programme self-sufficiency and cross-institutional scaling [18].

The results of our study present a critical knowledge deficit of SCS at a single centre. We advocate for the development of local and national SCS educational programs to facilitate the safe perioperative management of patients with SCS who may present to any surgical centre.

## Conclusions

There is a universal knowledge deficit in the assessment and management of perioperative SCS with a lack of training across all NCHD training grades. A brief, well-designed educational intervention can achieve extraordinary outcomes when targeted at a universal knowledge deficit. Combined with the evidence of universal pre-existing deficiency and the high impact of brief targeted teaching, this study constitutes a strong evidence base for curriculum change. The data are compelling, the need is universal, and the solution is proven to improve patient safety in the perioperative management of SCS.

## Appendices

Appendix A: pre-education survey

NCHD Awareness of Perioperative Management of Spinal Cord Stimulators (SCS)

1. What is your current training level?

Intern

SHO

Registrar

SpR

Other (please specify)

2. What specialty are you currently working in?

Anaesthesia

Surgery

Medicine

Emergency Medicine

Intensive Care

Other (please specify)

3. Prior to this survey, were you aware that patients may present for surgery with an implanted spinal cord stimulator (SCS)?

Yes

No

4. How confident are you in identifying a patient with a spinal cord stimulator during preoperative assessment?

Very confident

Somewhat confident

Neutral

Not confident

5. Are you aware that spinal cord stimulators generally require specific perioperative management (e.g., device deactivation, precautions with diathermy, MRI considerations)?

Yes

No

Unsure

6. Have you ever received formal teaching or training on the perioperative management of spinal cord stimulators?

Yes

No

7. If a patient with a spinal cord stimulator presented for surgery tomorrow, would you know where to find guidance on its perioperative management?

Yes

No

Unsure

8. Would you be interested in further teaching or guidelines regarding perioperative management of spinal cord stimulators?

Yes

No

Maybe

Appendix B: post-education survey 

NCHD Awareness of Perioperative Management of Spinal Cord Stimulators (SCS) in Ireland

1. What is your current training level?

Intern

SHO

Registrar

SpR

Other (please specify)

2. What specialty are you currently working in?

Anaesthesia

Surgery

Medicine

Emergency Medicine

Intensive Care

Other (please specify)

3. After the teaching session, how confident are you in identifying a patient with a spinal cord stimulator during preoperative assessment?

Very confident

Somewhat confident

Neutral

Not confident

4. After the teaching session, how confident are you in the perioperative management of a patient with a spinal cord stimulator?

Very confident

Somewhat confident

Neutral

Not confident

5. Which of the following is an important perioperative consideration in patients with a spinal cord stimulator? (Select all that apply)

The device may need to be switched off prior to surgery

Caution is required with diathermy/electrocautery

MRI compatibility should be checked

The device has no implications for perioperative care

Unsure

6. Do you now know where to find guidance on perioperative management of spinal cord stimulators?

Yes

No

Unsure

7. Has the teaching session improved your understanding of spinal cord stimulator management in the perioperative setting?

Significantly improved

Somewhat improved

No change

Unsure

8. Do you feel formal teaching on spinal cord stimulators should be included in NCHD education or induction programmes?

Yes

No

Unsure
